# The effect of US signalling and the US–CS interval on backward conditioning in mice

**DOI:** 10.1016/j.lmot.2014.08.002

**Published:** 2014-11

**Authors:** David J. Sanderson, Steven F. Cuell, David M. Bannerman

**Affiliations:** Department of Experimental Psychology, University of Oxford, South Parks Road, Oxford OX1 3UD, UK

**Keywords:** Learning, Conditioned Inhibition, Priming, Temporal Contiguity, Behaviour, External Inhibition

## Abstract

The effect of US signalling and the US–CS interval in backward conditioning was assessed in mice. For one group of mice the presentation of food was signalled by a tone and for another group, food was unsignalled. For half of the mice, within each group, the presentation of food preceded a visual cue by 10 s. For the other half, food was presented at the start of the visual cue (0-s US–CS interval), resulting in simultaneous pairings of these events. A summation test and a subsequent retardation test were used to assess the inhibitory effects of backward conditioning in comparison to training with a non-reinforced visual cue that controlled for the possible effects of latent inhibition and conditioned inhibition caused as a consequence of differential conditioning. In the summation test unsignalled presentations of the US resulted in inhibition when the US–CS interval was 10 s, but not 0 s. Signalled presentations of the US resulted in inhibition, independent of the US–CS interval. In the retardation test, independent of US signalling, a US–CS interval of 10 s failed to result in inhibition, but an interval of 0 s resulted in greater conditioned responding to the backward CS than the control CS. A generalisation decrement account of the effect of signalling the US with a 0-s US–CS interval, which resulted in reduced responding in the summation test and faster acquisition in the retardation test, is discussed.

Backward conditioning has been found to produce conditioned inhibition (e.g. Heth, 1976), suggesting that animals learn that the conditioned stimulus (CS) is a signal for the absence of the unconditioned stimulus (US) (Moscovitch & LoLordo, 1968). One factor affecting inhibitory backward conditioning is whether the occurrence of the US is anticipated or not. The inhibitory properties of a backward CS have been found to increase if the occurrence of the US is signalled by another cue (e.g. Dolan et al., 1985, Wagner and Terry, 1975, Williams and Overmier, 1988a). Moreover, US signalling can switch a backward CS from being an excitatory predictor of the US to being an inhibitor of the outcome (Dolan et al., 1985), suggesting that US signalling changes the nature of the association that is acquired.

Another factor that affects inhibitory backward conditioning is the interval between the US and the CS (e.g. Delamater et al., 2003, Hellstern et al., 1998, Maier et al., 1976, Romaniuk and Williams, 2000, Tanimoto et al., 2004). Increasing the US–CS interval has been found to increase conditioned inhibition (Hellstern et al., 1998, Romaniuk and Williams, 2000). However, if the US–CS interval is too long then condition inhibition fails to be acquired (Delamater et al., 2003, Hellstern et al., 1998, Maier et al., 1976, Tanimoto et al., 2004). Therefore, inhibitory backward conditioning occurs as an inverted-U shaped function of the US–CS interval.

While at face value US signalling and the US–CS interval appear to have the same qualitative effect on inhibitory backward conditioning, suggesting that they share a common psychological process (Wagner, 1981), they have not been directly tested within the same study. A problem in making comparisons across studies is that, in addition to the use of different species and training paradigms, different studies have often employed different control procedures, such as comparison of the backward CS to a novel CS (Dolan et al., 1985, Tanimoto et al., 2004), or to a CS that had been presented randomly in relation to the US (Williams & Overmier, 1988a). In addition, studies have used different tests of inhibition (e.g. summation test (Dolan et al., 1985, Hellstern et al., 1998, Romaniuk and Williams, 2000, Williams and Overmier, 1988a), retardation test (Hellstern et al., 1998), preference test (Tanimoto et al., 2004) and Pavlovian-to-instrumental transfer test (Delamater et al., 2003)). Moreover, only the study by Hellstern et al. (1998) employed both summation and retardation tests, leaving the possibility that in other studies positive results in either summation or retardation tests may potentially be open to alternative accounts, such as attentional explanations of performance and/or learning (Papini and Bitterman, 1993, Rescorla, 1969).

The aim of the present study was to assess US signalling and the US–CS interval within the same study, using a common control procedure and tests of conditioned inhibition. Appetitive conditioning in mice was assessed and US signalling and the US–CS interval were manipulated in a between-subjects factorial design (see Table 1). Conditioned inhibition was first measured using a summation test, and then with a retardation test. Half of the mice received backward conditioning (with stimulus B), in which the US was signalled by a CS (signalled). The other half of mice received backward conditioning, but the US was not signalled by a CS (unsignalled). Within each of the signalled and unsignalled groups, half of the mice were trained with a US–CS interval of 0 s (0-s delay) and the other mice were trained with a US–CS interval of 10 s (10-s delay). Whereas a 10-s delay will likely result in the consumption of the US before the presentation of the CS, a 0-s delay will result in the simultaneous presentation of the US and CS. Mice also received training with a control stimulus (C) that was not paired with the US and was presented as often as B. Stimulus C controlled for a number of possible effects that are not specific to the effect of US signalling and the US–CS interval on conditioned inhibition. First, conditioned inhibition may be caused by negative prediction error (Wagner & Rescorla, 1972) due to the context becoming excitatory throughout training and the backward CS signalling the absence of the US (see Chang et al., 2003, Williams et al., 1990, Williams and Overmier, 1990). The extent to which negative prediction error affects performance will be equal for both B and C. Second, it is possible that as a consequence of exposure during backward conditioning that a backward CS may undergo latent inhibition, and may subsequently be retarded in acquisition of conditioned responding for reasons other than true conditioned inhibition (Rescorla, 1971). Due to B being presented as often as C, B would not be expected to be more latently inhibited than C. It was predicted that if US signalling and the US–CS interval both alter processing of the US such that B forms an inhibitory association, then both manipulations should affect performance on the summation and retardation tests in a qualitatively similar manner. The factorial experimental design also allowed assessment of the possible additive effects of both US signalling and the US–CS interval on conditioned inhibition.

**Table 1:** Design of backward conditioning procedure. Food-B signifies simultaneous presentation of food and the backward CS (0 s delay), whereas Food → B signifies that food preceded the backward CS (10 s delay). C = control CS, N = noise CS.

Group	Stage 1 – signal training	Stage 2 – backward conditioning	Stage 3 – summation test	Stage 4 – retardation test
Unsignalled – 0 s delay	Tone → Food	Food-B	N N-B N-C	B → Food C → Food
		C		
		N → Food		

Unsignalled – 10 s delay	Tone → Food	Food → B		
		C		
		N → Food		

Signalled – 0 s delay	Tone → Food	Tone → Food-B		
		C		
		N → Food		

Signalled – 10 s delay	Tone → Food	Tone → Food → B		
		C		
		N → Food		

Mice were chosen as subjects for the study in order to develop behavioural procedures that may be used to take advantage of recent developments in genetic technology in mice for studying the neural basis of learning and memory. As noted by others (Bonardi et al., 2010, Gonzalez et al., 2013), there are a large number of well-established behavioural procedures for studying aspects of learning and memory in rats, but as yet the number of effective procedures in mice is limited. Thus, development of behavioural procedures in mice will increase the scope of manipulations for examining the neural basis of cognition. Furthermore, genetic manipulations in mice have revealed psychological dissociations not previously observed with other manipulations (Bannerman et al., 2014, Sanderson and Bannerman, 2012). Therefore, the development of behavioural procedures that can be used for genetically modified mice will also aid the psychological analysis of behaviour.

Backward conditioning has often been studied using aversive, fear conditioning procedures (e.g. Chang et al., 2003, Dolan et al., 1985, Heth, 1976, Maier et al., 1976, McNish et al., 1997, Moscovitch and LoLordo, 1968, Romaniuk and Williams, 2000, Siegal and Domjan, 1971, Tanimoto et al., 2004, Wagner and Terry, 1975, Williams and Overmier, 1988a), because it is possible to have greater temporal control over the events (US and CS) than compared to appetitive conditioning, in which experience of the US is dependent on the behaviour of the animal. However, inhibitory backward conditioning has been shown successfully using appetitive procedures (Delamater et al., 2003), and the inhibitory effects of backward conditioning have been suggested to contribute to trial spacing effects in Pavlovian conditioning (Ewing, Larew, & Wagner, 1985) and discrimination learning (Honey, 1996) procedures that have used food as the US. The present experiment used an appetitive procedure, in part, to test the generality of the US signalling and US–CS interval effects found in fear conditioning procedures, but also because appetitive procedures have particular advantages over aversive procedures (Bonardi et al., 2010). For example, conditioning, in which shock is used as the CS, tends to be rapid compared to appetitive procedures, and may, in addition, result in strong context conditioning. Rapid conditioning may reduce the potential for observing the effects of US signalling and the US–CS interval, due to learning reaching similar asymptotic levels in all conditions. Strong context conditioning may diminish the impact of signalling the US (because the context also signals the US). It may also increase the likelihood that both the backward and control CSs become conditioned inhibitors because of negative prediction error. This may reduce the ability to detect differences between cues that are caused by US signalling and the US–CS interval.

## Methods

### Subjects

Thirty-two female C57BL/6J/Ola mice obtained from Harlan OLAC Ltd (Oxon, UK) were used. Mice were caged in groups of eight, in a temperature controlled housing room (light-dark cycle: 0700–1900). The mice were approximately 10 weeks old and weighed a mean of 18.4 g (range = 16.1–19.8 g) at the start of testing. Mice were initially allowed free access to food, but one week prior to training the weights of the mice were reduced, by receiving a restricted diet, and then subsequently maintained at 85% of their free-feeding weight. Mice were tested during the light period between 10 am and 2 pm. Throughout testing mice had ad libitum access to water in their home cages.

### Apparatus

Eight identical operant chambers (15.9 cm × 14.0 cm × 12.7 cm; ENV-307A, Med Associates), enclosed in sound-attenuating cubicles (ENV-022MD, Med Associates), controlled by Med-PC IV software were used. The front and back walls and the ceiling of each chamber were made from clear Perspex, and the sidewalls were made from aluminium. The floor was a grid of stainless steel rods (0.32 cm diameter), each separated by 0.79 cm. Sucrose pellets (20 mg TestDiet, ETH) could be dispensed into a magazine (2.9 cm × 2.5 cm × 1.9 cm; ENV-303M, Med Associates) located in the centre of one of the sidewalls. Breaks in an infrared beam (ENV-303HDM, Med Associates) across the bottom of the entrance to the magazine were used to measure the number of magazine head entries at a resolution of 0.1 s. White noise and a pure tone (3 kHz), each at 75 dB (∼10 dB above background noise), generated by an audio generator (ANL-926, Med Associates) could be emitted from a speaker (ENV-324M, Med Associates) located at the top right corner of the wall opposite the magazine. A 28 V, 100 mA house light (ENV-315M, Med Associates) was located next to the speaker in the centre of the wall. Presentation of the house light resulted in illumination of the chamber. Two LEDs (ENV-321M, Med Associates) were positioned to the left and the right, above the magazine. Presentation of the LEDs resulted in limited, localised illumination. A fan (ENV-025AC) was positioned above the left LED and was turned on during sessions.

### Procedure

#### Stage 1 – tone training

All mice received eight sessions (one per day) of conditioning to establish a 10-s presentation of a tone as a predictor of food. The tone was presented six times per session with a variable 370-s inter-trial interval (range = 143–846 s, based on a Fleshler-Hoffman distribution (1962)). At the termination of the tone a single sucrose pellet was dispensed. The number of magazine entries that mice made was recorded during the tone presentation and the 10-s period prior to the tone.

#### Stage 2 – backward conditioning

The day after Stage 1, mice received two eight-session blocks of backward conditioning, one session per day, with a 48-hr interval between each eight-session block. Each session consisted of six trials of a 10-s presentation of white noise (N) that terminated with the presentation of a sucrose pellet, six backward conditioning trials with a presentation of a light (B), and six trials with a presentation of a different light (C) that was not paired with sucrose pellets.

The effects of signalling the US and the US–CS interval were manipulated in a 2 (US signal: unsignalled, signalled) × 2 (delay: 0 s, 10 s) factorial design. Mice were randomly assigned to one of the four groups: unsignalled – 0-s delay, unsignalled – 10-s delay, signalled – 0-s delay, and signalled – 10-s delay (*N* = 8 per group). Mice in the signalled conditions received backward conditioning trials in which the US was immediately preceded by the tone. Thus, at the termination of the tone a pellet was dispensed. For mice in the unsignalled condition the US was not preceded by the tone and they received no further presentations of the tone. For mice in 0-s delay conditions the US was presented immediately at the onset of B, whereas it was presented 10 s prior to B for mice in the 10-s delay conditions.

The stimuli used as B and C were presentation of a 10-s house light, and a 10-s presentation of the two flashing LEDs (1 s on/1 s off). The allocation of stimuli as B and C was counterbalanced within each group. Trials with N, B or C were separated by a fixed interval of 120 s that commenced at the termination of one CS (N, B or C) and ended at the onset of another CS (N, B or C). The order of the different trial types was random with the constraint that there were equal numbers of each trial type every block of six trials. For all trials responding was recorded during the stimuli and the 10-s period prior to the presentation of the stimuli. In addition, for B trials, responding was recorded in 10-s bins for the 30-s period before presentation of the light.

#### Stage 3 – summation test

Mice received two sessions in which a summation test was conducted. The first test session was conducted 24 hr after the first eight-session block of backward conditioning, and the second was conducted 24 hr after the second eight-session block of backward conditioning. The test sessions consisted of three different trial types: (i) trials in which the noise was presented alone (N), trials in which the noise was presented in compound with B (N-B), and trials in which the noise was presented in compound with C (N-C). There were six trials of each trial type per session. All trials were conducted in extinction (i.e., sucrose pellets were no longer presented). The order of trial type was pseudo-random, with an equal number of each trial type every block of three trials. Within each block of three trials the order of the N-B and N-C trials was counterbalanced within each group. Trials were separated by a fixed interval of 120 s, and responding was recorded in 2-s blocks during trials and the 10 s prior to a trial.

#### Stage 4 – retardation test

The day after the final summation test session, mice received a retardation test conducted over 12 sessions, one session per day. Each session consisted of six trials with B and six with C. A sucrose pellet was dispensed at the termination of both stimuli. Trials were separated by a variable interval of 180 s (range = 125–288 s, based on a Fleshler-Hoffman distribution (1962)). The order of the trials was random with the constraint that there was an equal number of each trial type within every block of four trials. Responding was recorded in 2-s blocks during the stimuli and in the 10 s prior to a stimulus presentation.

### Statistical analyses

The number of magazine entries is reported as responses per min (RPM). Data were analysed using multifactorial analysis of variance (ANOVA). Significant interactions were analysed using simple main effects analysis using the pooled error term from the original ANOVA, or separate ANOVAs for repeated measures with more than two levels.

## Results

### Stage 1 – tone training

Conditioning progressed over training such that during the last two-session block mice made a greater number of responses during the tone (mean = 16.88 RPM ± 2.33 SEM) than in the pre-CS period (mean = 3.50 RPM ± 0.52 SEM). There was a significant block by period (CS vs. pre-CS) interaction (*F*(3, 84) = 38.17, *p* < 0.001). Simple main effects analysis of the interaction revealed that mice responded at a significantly greater level during the tone than during the pre-CS period from the third block onwards (*F* values > 11, *p* values < 0.05). Mice assigned to the different delay and signalled conditions did not significantly differ (*F* values < 1), and there were no other significant interactions of factors (*p* values > 0.8).

### Stage 2 – backward conditioning

Across sessions 8 and 16, the two sessions prior to the summation tests, mice made a greater number of responses during the N trials (mean = 36.12 RPM ± 1.43 SEM) than during the C trials (mean = 1.96 RPM ± 0.30 SEM). In contrast, responding during the pre-CS periods was similar (N, mean = 2.88 RPM ± 0.39 SEM; C, mean = 3.05 RPM ± 0.39). A 2 (session: 8, 16) × 2 (trial type: N, C) × 2 (period: pre-CS, CS) × 2 (US signal: unsignalled, signalled) × 2 (delay: 0 s, 10 s) ANOVA confirmed these observations. There was a significant effect of trial type (*F*(1,28) = 353.03, *p* < 0.001) and a significant effect of period (*F*(1,28) = 449.88, *p* < 0.001), and these factors significantly interacted (*F*(1,28) = 461.82, *p* < 0.001). Simple main effects analysis showed that mice made significantly more responses during N than in the pre-CS period (*F*(1,28) = 476.07, *p* < 0.001), but this was not true for C (*F*(1,28) = 1.36, *p* = 0.25). There were no other significant main effects or interactions (*p* values > 0.1).

**Fig. 1:**
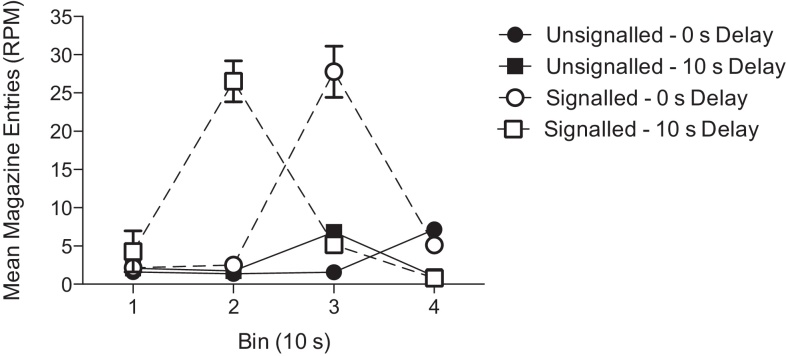
Mean magazine entries (RPM) during the backward conditioning trials (bin 4) and the three 10-s time bins prior to the backward conditioning trials (bins 1–3). The data are the means of sessions 8 and 16 of training, the two sessions prior to the two summation test sessions. Error bars indicate ± S.E.M.

The level of responding on the backward conditioning trials collapsed across sessions 8 and 16, the two sessions prior to the summation tests, is shown in Fig. 1. In addition, Fig. 1 shows the responses during the three 10-s time bins prior to the backward conditioning trial. A 2 (session: 8, 16) × 4 (time bin) × 2 (US signal: unsignalled, signalled) × 2 (delay: 0 s, 10 s) ANOVA revealed a significant effect of time bin (*F*(3,84) = 17.12, *p* < 0.001), a significant effect of US signal (*F*(1,28) = 62.44, *p* < 0.001) and significant interactions between time bin and delay (*F*(3,84) = 34.05, *p* < 0.001), time bin and US signal (*F*(3,84) = 18.19, *p* < 0.001), and time bin, delay and US signal (*F*(3,84) = 45.15, *p* < 0.001). To analyse the three-way interaction, separate ANOVAs were conducted for each time bin. On the first time bin, in which no stimuli were presented, there was no significant interaction between delay and US signal (*F* < 1, *p* > 0.4), and the main effects of delay (*F*(1,28) = 2.58, *p* > 0.1) and US signal (*F*(1,28) = 1.48, *p* > 0.2) were not significant. On the second time bin in which the tone was presented for the signalled – 10-s delay group there was a significant delay by US signal interaction (*F*(1,28) = 53.15, *p* < 0.001). Simple main effects analysis showed that the signalled – 10-s delay group made significantly more responses than the signalled 0-s delay group (*F*(1,28) = 116.80, *p* < 0.001), but there was no significant difference between the unsignalled groups (*F* < 1, *p* > 0.6). In addition the signalled – 10-s delay group made significantly more responses than the unsignalled – 10-s delay group (*F*(1,28) = 115.46, *p* < 0.001), but there was no significant difference between the 0-s delay groups (*F* < 1, *p* > 0.6). On the third time bin in which the US was presented for the signalled – 10-s delay group, the tone was presented for the signalled – 0-s delay group, and the US was presented for the unsignalled – 10-s delay group, there was a significant delay by US signal interaction (*F*(1,28) = 45.54, *p* < 0.001). Simple main effects analysis showed that the signalled – 0-s delay group made significantly more responses than the signalled – 10-s delay group (*F*(1,28) = 47.70, *p* < 0.001), and the unsignalled – 10-s delay group made significantly more responses, presumably due to collecting the US, than the unsignalled – 0-s delay group (*F*(1,28) = 6.95, *p* = 0.013). In addition the signalled – 0-s delay group made significantly more responses than the unsignalled – 0-s delay group (*F*(1,28) = 74.63, *p* < 0.001), but there was no significant difference between the 10-s delay groups (*F* < 1, *p* > 0.3). On the final block in which B was presented for all groups, and the US was presented for the 0-s delay groups, there was no significant interaction between delay and US signal (*F*(1,28) = 1.58, *p* > 0.2). However, there was a significant effect of delay (*F*(1,28) = 94.18, *p* < 0.001), presumably due to mice in the 0-s delay condition collecting the food pellet.

### Stage 3 – summation test

**Fig. 2:**
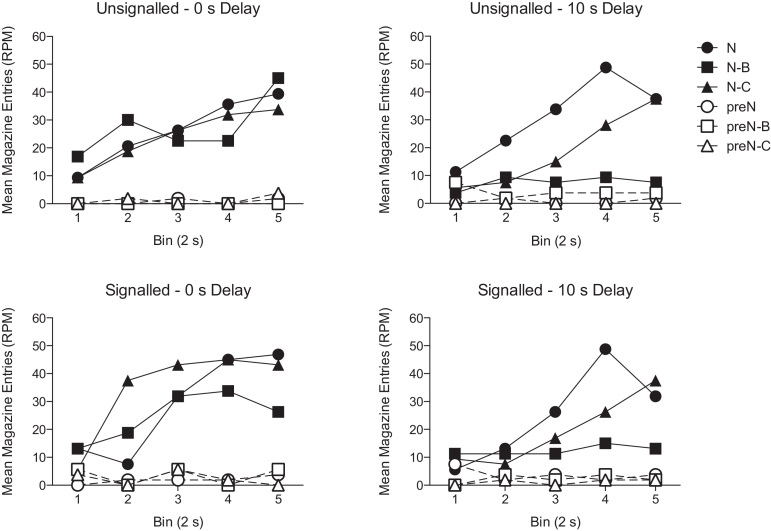
Summation test performance on N, N-B and N-C trials and for their respective pre-CS periods. Magazine entries (RPM) are shown for the CSs and the pre-CS periods in 2-s bins for the duration of the stimulus.

**Fig. 3:**
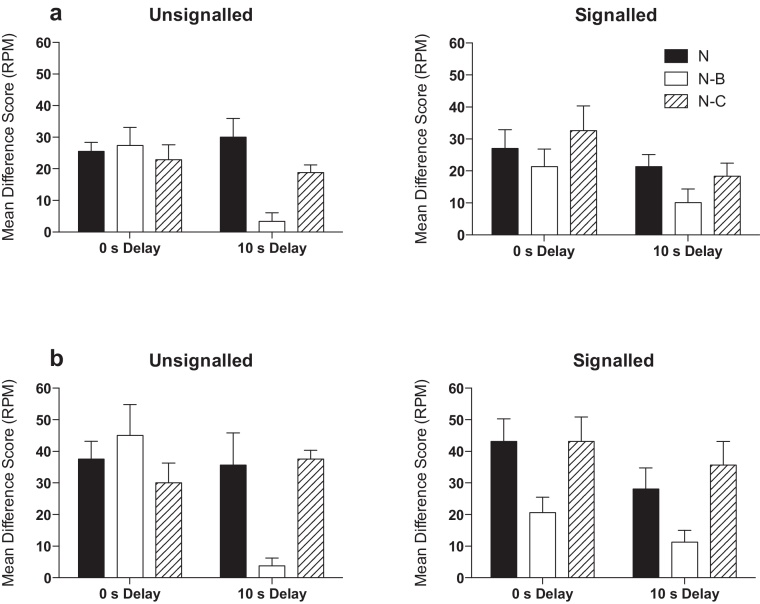
Summation test performance shown as difference scores (CS minus pre-CS; RPM). Panel a shows the results collapsed across the 10-s duration of the test trials. Panel b shows the results restricted to the last 2 s of each trial type. Error bars indicate S.E.M.

During the summation test sessions conditioned responding extinguished rapidly across trials and consequently performance on the different trial types increasingly converged. Therefore, analysis of performance during the different trial types was limited to the mean performance on the first trial of each test session, of each trial type (see Fig. 2). Responses were converted to difference scores in which pre-CS responses were subtracted from CS responses (see Fig. 3a). Importantly, there were no significant effects or interactions of trial type, US signalling, or delay on pre-CS responding (overall mean = 1.78 RPM ± 0.41, *p* values > 0.09).

All groups showed a strong increase in magazine entries during N. The unsignalled – 0-s delay group showed a similar level of responding during both the compound trials, N-B and N-C, in comparison to N. The unsignalled – 10-s delay group showed a different pattern of performance, responding at a lower rate during N-B than during N and N-C. The pattern of performance for the signalled groups was similar to that of the unsignalled – 10-s delay group, but the reduction in responding to N-B was numerically lower. A 3 (trial type: N, N-B, N-C) by 2 (US signal: unsignalled, signalled) by 2 (delay: 0 s, 10 s) ANOVA revealed a significant effect of trial type (*F*(2,56) = 8.43, *p* = 0.001), and a significant effect of delay (*F*(1,28) = 5.75, *p* = 0.023). The effect of delay significantly interacted with trial type (*F*(2,56) = 5.30, *p* = 0.008), and there was a three-way interaction between delay, trial type and US signal (*F*(2,56) = 3.17, *p* = 0.05). There were no other significant main effects or interactions (*p* values > 0.29).

The three-way interaction was explored by conducting separate ANOVAs for the signalled and unsignalled groups. For the unsignalled groups, there was a significant effect of trial type (*F*(2,28) = 6.25, *p* = 0.006) that significantly interacted with delay (*F*(2,28) = 8.67, *p* = 0.001). The main effect of delay was not significant (*F*(1,14) = 2.98, *p* = 0.106). Simple main effects analysis of the interaction showed that there was a significant effect of delay for trial type N-B (*F*(1,14) = 14.58, *p* = 0.002), but not for N (*F*(1,14) < 1, *p* > 0.5) or N-C (*F*(1,14) < 1, *p* > 0.4). Also, there was a significant effect of trial type for the 10-s delay group (*F*(2,13) = 16.07, *p* < 0.001), but not for the 0-s delay group (*F*(2,13) < 1, *p* > 0.5). Post hoc analyses of the effect of trial type for the 10-s delay group, using the Bonferroni correction, revealed that responding during N-B was significant lower than during N (*p* = 0.001) and N-C (*p* = 0.002). Responding during N and N-C did not significantly differ (*p* = 0.349).

The analysis for the signalled groups revealed a significant effect of trial type (*F*(2,28) = 3.69, *p* = 0.038), but no other significant effects or interactions (*p* values > 0.1). Post hoc analyses of the effect trial type, using the Bonferroni correction, failed to reveal any significant pairwise differences (*p* values > 0.09).

The failure to find to clear inhibitory effects of backward conditioning in the signalled groups may reflect a lack of sensitivity in measuring performance across the whole duration of trial. Inspection of the data revealed that responding was suppressed during N-B in comparison to responding during N, only in the latter portions of the compound stimulus (see Fig. 2). This result is similar to the findings of Romaniuk and Williams (2000) who found that the latter portions of a backward CS were more inhibitory than at the beginning of the CS. While it may be the case that the latter portions of B were more inhibitory, a simple, alternative explanation of our results is that B may have appeared more inhibitory across the duration of the CS because magazine approach behaviour increased during N (see Holland, 1977). This would have meant that there was more opportunity for measuring suppression towards the end of the CS. Given this observation, analysis of the rates of responding towards the end of the CS may provide a more sensitive measure. Thus, identical analyses limited to the last 2 s of each trial type revealed an overall similar pattern of results (three-way interaction, *F*(2,56) = 6.187, *p* = 0.004, see Fig. 3b), but now post hoc analyses of the effect of trial type, using the Bonferroni correction, for the signalled groups revealed that responding during N-B was significantly lower than during N (*p* < 0.001) and N-C (*p* < 0.001), but that there was no significant difference between N and N-C (*p* = 0.506).

Using the data from the last 2 s of CS responding (Fig. 3b), additional ANOVAs were conducted in order to determine whether the different pattern of results found with the signalled and unsignalled groups reflected a significant effect of US signalling on the different trial types. There was no significant US signal by trial type interaction when the US–CS interval was 10 s (*F*(2,28) = 1.13, *p* = 0.34), but there was a significant interaction when the interval was 0 s (*F*(2,28) = 6.26, *p* = 0.006). Simple main effects analysis revealed that there was a significant effect of trial type when the US was signalled (*F*(2,13) = 5.09, *p* = 0.023), but not when unsignalled (*F*(2,13) = 1.63, *p* = 0.23). In addition signalling the US reduced responding to N-B (*F*(1,14) = 4.95, *p* = 0.043), but not N (*F* < 1) or N-C (*F*(1,14) = 1.72, *p* = 0.21).

### Stage 4 – retardation test

The rates of responding during the retardation test were converted to difference scores in which the number of responses during the pre-CS periods was subtracted from the CS periods. Scores greater than zero indicated that mice responding more during the CS than in the pre-CS period.

**Fig. 4:**
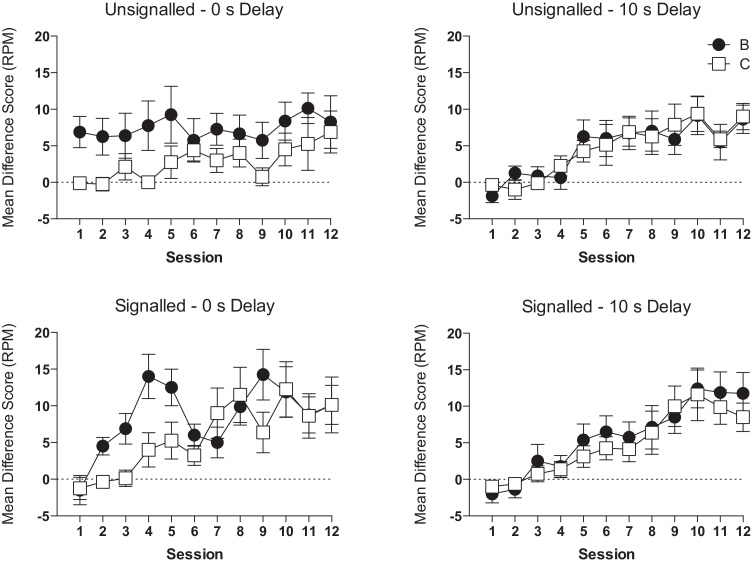
Retardation test performance for B and C. Responding is shown as the mean difference score of magazine entries (CS minus pre-CS; RPM). The dashed line indicates chance performance. Error bars indicate ± S.E.M.

Fig. 4 shows the acquisition of conditioned responding over the 12 sessions of training. At the start of training, group unsignalled – 0-s delay showed greater responding to B than C. However, as conditioned responding was acquired to C, this difference was reduced over training. The signalled – 0-s delay group showed faster acquisition of conditioned responding to B than to C, but the difference was reduced over training. There was little difference in the rate of acquisition of conditioned responding to B and C for both of the 10-s delay groups.

A 2 (trial type: B, C) × 12 (session) × 2 (US signal: unsignalled, signalled) × 2 (delay: 0 s, 10 s) ANOVA of the difference scores showed a significant effect of trial type (*F*(1,28) = 15.81, *p* < 0.001) and session (*F*(11,308) = 17.28, *p* < 0.001). There was a significant interaction between trial type and delay (*F*(1,28) = 9.02, *p* = 0.006), trial type and session (*F*(11,308) = 1.95, *p* = 0.033) and session and US signal (*F*(11,308) = 2.06, *p* = 0.023). There was a three-way, trial type by delay by session interaction (*F*(11,308) = 2.89, *p* = 0.001). There were no other significant main effects or interactions (*p* values > 0.05). Simple main effects analysis of the session by US signal interaction showed that mice in the signalled groups responded at a significantly higher rate than the unsignalled groups on session 1 (*F*(1,28) = 5.279, *p* = 0.029), but not thereafter (largest *F*(1,28) = 3.98, *p* > 0.05). Separate ANOVAs confirmed that there was a significant effect of session for signalled groups (*F*(11,154) = 13.07, *p* < 0.001) and unsignalled groups (*F*(11,54) = 5.44, *p* < 0.001).

The three-way, trial type by delay by session interaction was analysed by conducting separate ANOVAs for the 0-s and 10-s delay groups. For the 0-s delay group, there was a significant trial type by session interaction (*F*(11,54) = 3.67, *p* < 0.001). Simple main effects analysis of the interaction revealed that mice showed significantly greater responding to B than to C on sessions 1–5 and on session 9 (smallest *F*(1,14) = 7.29, *p* = 0.017). There were no significant differences on the remaining sessions (largest *F*(1,14) = 2.00, *p* > 0.15). Separate ANOVAs for each trial type confirmed that there was a significant effect of session for B (*F*(11,154) = 3.39, *p* < 0.001) and C (*F*(11,54) = 5.99, *p* < 0.001). For the 10-s delay group, there was a significant effect of session (*F*(11,154) = 15.25, *p* < 0.001), no significant effect of trial type (*F*(1,14) < 1, *p* > 0.3), and no significant trial type by session interaction (*F*(11,154) < 1, *p* > 0.6).

A similar 2 (trial type: B, C) × 12 (session) × 2 (US signal: unsignalled, signalled) × 2 (delay: 0 s, 10 s) ANOVA of the rates of responding during the pre-CS periods (overall mean = 2.58 RPM ± 0.35 SEM), also showed a significant trial-type by session by delay interaction (*F*(11,308) = 2.56, *p* = 0.004), but no other significant main effects or interactions (*p* values > 0.08). The three-way interaction was analysed by conducting separate ANOVAs for the 0-s and 10-s delay groups. There was no significant trial-type by session interaction for the 10-s delay groups (*F*(11,154) = 1.06, *p* > 0.3), but there was for the 0-s delay groups (*F*(11,154) = 2.27, *p* = 0.014). However, simple main effects analysis failed to reveal a significant effect of trial type on any session (largest *F* value, session 7, *F*(1,14) = 3.91, *p* > 0.06), and separate ANOVAs for each trial type failed to reveal a significant effect of session for B (*F*(11,154) < 1, *p* > 0.4) or C (*F*(1,154) = 1.20, *p* > 0.2). Therefore, it is unlikely that the differences in responding to B and C between the groups reflect differences in baseline, pre-CS levels of responding.

In the summation test, it was found that the unsignalled – 0-s delay group showed a significantly different pattern of performance from the unsignalled – 10-s delay group, with the 10-s delay group showing inhibition, but not the 0-s delay group. The results of the retardation test imply that the pattern of performance significantly differed between these groups, with the 0-s delay group, but not the 10-s delay group, showing greater responding to B than to C. This pattern was confirmed to be the case by a significant delay by trial type by session interaction (*F*(11,154) = 2.83, *p* = 0.002), when the ANOVA was restricted to the unsignalled groups.

Furthermore, the results of the summation test failed to reveal a significant effect of US–CS interval when the US was signalled. However, the results of the retardation test imply that the pattern of performance significantly differed between these groups, with the 0-s delay group, but not the 10-s delay group, showing greater responding to B than to C. This finding was confirmed to be the case by a significant delay by trial type interaction (*F*(1,14) = 6.89, *p* = 0.02), when the ANOVA was restricted to the signalled groups.

## Discussion

The aim of the study was to test whether US signalling and US–CS interval manipulations in backward conditioning have the same qualitative effect on learning, using summation and retardation tests of conditioned inhibition. The collective results fail to provide evidence that signalling the US and extending the US–CS interval have the same qualitative effect, with the different manipulations producing different patterns of results on the two tests. A potential problem in examining the effects of backward conditioning is the change in the conditions during training and at the test of learning, leading to generalisation decrement. We will subsequently discuss whether this confounding factor was greatest for the signalled – 0-s delay group, resulting in opposing inhibitory and excitatory effects in the summation and retardation tests, respectively.

When the US–CS interval was 10 s, the backward CS passed the summation test regardless of whether the US had been signalled or not. However, in the retardation test, these groups failed to show inhibition with conditioning progressing at a similar rate for the backward and control CSs. When the US–CS interval was 0 s there was a different pattern of performance. The signalled group passed the summation test, but the unsignalled group did not. However, in the retardation test, both groups showed greater conditioning to the backward CS than to the control CS. This effect is the opposite of the effect expected if the backward CS was a conditioned inhibitor.

Using Rescorla's criteria (Rescorla, 1969) for conditioned inhibition, because no one group passed both the summation and retardation test, it is not possible to claim that the procedures employed in the present experiment yielded conditioned inhibition. However, there were differences between the groups on different tests that make it possible to deduce that the manipulations, under certain conditions, affected the nature of associative learning, suggesting that conditioned inhibition may be inferred. Thus, when the US was unsignalled, a 10-s US–CS interval resulted in inhibition in the summation test, whereas a 0-s interval did not. However, a 0-s US–CS interval resulted in excitation in the retardation test, but a 10-s interval did not. This pattern of effects suggests that the different US–CS intervals produced opposite effects on backward conditioning. Given this difference it may be concluded that, although the 10-s interval group failed to pass the retardation test, increasing the US–CS interval facilitated the formation of an inhibitory association. This point will be discussed further later on in the discussion.

The most surprising results were that, when the US–CS interval was 0 s, signalling the US resulted in the backward CS passing the summation test but then subsequently showing greater excitatory conditioning than the control CS in the retardation test. Thus, the backward CS appeared inhibitory in the summation test and excitatory in the retardation test. Although it has been claimed that CSs can acquire both excitatory and inhibitory properties (Williams and Overmier, 1988b, Williams et al., 1992), in this instance the opposite effects of signalling the US with a 0-s US–CS interval may be explained in simpler terms. It is possible that, when the backward CS was presented in compound with the excitatory CS in the summation test, there was significant generalisation decrement between the conditions during training and at test, which meant that the CS was treated as being novel in comparison to the control CS. For example, during training the CS had always been experienced in the context of a prior presentation of the tone CS and the presentation of the US, whereas in the summation test it was now presented in the absence of the tone and US and now in the presence of the noise CS. Generalisation decrement between training and test may result in dishabituation of unconditioned responding and in response competition, and therefore, potentially a form of external inhibition as opposed to conditioned inhibition. For example, renewed orienting to the backward CS may have interfered with magazine approach behaviour. Furthermore, generalisation decrement would lead to the backward CS being perceived as novel in comparison to the control light. Therefore, the backward CS would condition more readily in the retardation test due to the control CS being latently inhibited. The pattern of performance of the signalled – 0-s delay group in summation and retardations tests is consistent with the notion that the CS received a high degree of attention rather than being a conditioned inhibitor (Rescorla, 1969).

In contrast to the signalled – 0-s delay group, the groups trained with a 10-s US–CS interval did not show excitation in the retardation test. However, the groups trained with a 10-s US–CS interval passed the summation test. This dissociation suggests that it is not necessary to appeal to an external inhibition account and/or increased attention account of the performance of the two groups trained with a 10-s US–CS interval in the summation test. Therefore, the summation test performance in the groups trained with a 10-s US–CS interval may instead reflect true conditioned inhibition. If this was the case, then why did these groups fail the retardation test? One possibility is that the use of an equally exposed, nonreinforced CS (C) was an overly conservative control procedure for the retardation test. It is possible that the control CS was latently inhibited, but the backward CS was not. Given that the potential effect of conditioned inhibition for the backward CS may be equal to, or even less than the effect of latent inhibition for the control CS, then it may not be likely that the backward CS would acquire conditioned responding less rapidly than the control CS in the retardation test. Another possibility is that the order of the summation and retardation tests reduced the sensitivity of the retardation test. However, sequential summation and retardation tests have been used successfully in other studies (Bonardi et al., 2010) and any test order effect would likely work against the positive retardation test findings for the 0-s delay groups. Regardless of whether or not the failure to pass the retardation test was due to a floor effect or reduced sensitivity, the fact that the groups trained with a 10-s US–CS interval did not show excitation in the retardation test rules out a potential non-specific account of inhibitory effects in these groups, suggesting that backward conditioning resulted in an inhibitory CS-US association.

A dissociation was also found between the signalled and unsigalled groups trained with a 0-s US–CS interval. Whereas, both the signalled and unsignalled groups showed greater conditioned responding to the backward CS than the control CS in the retardation test, only the group for which the US was signalled passed the summation test. This finding raises the possibility that the excitatory effect of backward conditioning in the retardation test was caused by different factors in the two groups. As previously discussed, it is likely that performance in the signalled – 0-s delay group was driven by increased attention to the backward CS caused by generalisation decrement between training and test. However, as the unsignalled – 0-s delay group did not show suppression in the summation test, it seems unlikely that the excitatory effect of backward conditioning in the retardation test in this group was due to increased attention. One possibility is that for the unsignalled – 0-s delay group, backward conditioning resulted in excitatory conditioning. Given that there is variability in the conditions in which two excitatory CSs produce summation (Aydin and Pearce, 1995, Aydin and Pearce, 1997, Myers et al., 2001, Pearce et al., 1997, Pearce et al., 2002), the retardation test may have been a more sensitive means of detecting excitation. Nonetheless, the unsignalled – 0-s delay group showed a trend for enhanced responding rather than a suppressive effect in the summation test. Furthermore, the unsignalled – 0-s delay group showed superior conditioned responding to the backward CS that was evident on the first session of the retardation test, whereas this effect did not emerge until the second session in the signalled – 0-s delay group.

Collectively, the effect of signalling the US across the two US–CS intervals poses a conundrum. While signalling the US with a 0-s US–CS interval increased attention to the backward CS, signalling the US with a 10-s US–CS interval was without effect. It is possible that when the US–CS interval was 0 s, signalling the US interfered with the formation of an excitatory association. This possibility could be due to the signal reducing the degree to which the US is processed (Wagner, 1981). However, when the US–CS interval was 10 s, signalling the US did not stop the CS from becoming inhibitory. This raises the possibility that when the US–CS interval was 0 s, signalling the US did not interfere with US processing, but that the effect of generalisation decrement masked the detection of any potential excitatory or inhibitory association. The ability to measure excitatory or inhibitory learning in any of the groups may have been tempered by potential generalisation decrement between training and test. However, signalling the US may have caused greater generalisation decrement when the US–CS interval was 0 s than 10 s because there was a greater change in the total pattern of stimulation at the time of the CS presentation.

While it may not be possible to conclude that signalling the US resulted in increasing the formation of inhibitory associations, the results do suggest that signalling the US can result in a CS appearing inhibitory in a summation test for reasons other than true conditioned inhibition. This is in contrast to other findings that have suggested that US signalling does increase inhibitory associative learning (Dolan et al., 1985, Williams and Overmier, 1988a). In the study by Dolan et al. (1985), signalling the US in backward conditioning was assessed with a summation test in which the inhibitory effects of the backward CS were compared to a novel stimulus. It was found that when the US was signalled, the backward CS suppressed conditioned responding to a greater extent than the novel stimulus. It is unlikely that generalisation decrement leads to greater attention being paid to the backward CS than the novel stimulus, suggesting that, in contrast to the present study, an attentional account may not apply to the results of Dolan et al. (1985). This argument implies that the rat fear conditioning procedure used by Dolan et al. (1985) yields different effects than the appetitive goal tracking procedure employed with mice in the present study. However, it should be noted that there are other differences that may lead to the outcomes in the two studies. For example, in the Dolan et al. (1985) study, the novel control stimulus caused a high degree of suppression of conditioned responding, whereas in the present study the control stimulus failed to cause any discernable amount of suppression. Therefore, the effects of backward conditioning in the two experiments were compared on different baselines.

It is possible that the inhibitory effect of signalling the US in the study by Williams and Overmier (1988a) may have been due to increased attention caused by generalisation decrement. The inhibitory effect of signalling the US was compared only to the effect of unsignalled US presentations. The change in the total pattern of stimulation between training and test would have been greater for the signalled group than for the unsignalled group. However, Williams and Overmier (1988a) also found conditions in which signalling the US did not produce greater inhibition than unsignalled USs. For example, there was an effect of signalling the US only when trials were distributed across sessions rather than occurring in a single session. Williams and Overmier were able to explain this pattern of results in terms of influences on inhibitory conditioning. In contrast, generalisation decrement does not readily account for this dissociation.

Overall, although there were no significant inhibitory effects in the retardation test, the results suggest that the US–CS interval in backward conditioning changes the nature of the association that is acquired (i.e. excitatory or inhibitory). There was little evidence to support the role of US signalling in inhibitory conditioning, but this may be due to problems in detecting inhibition due to the generalisation decrement between training and test. Therefore, while the results do not provide direct evidence against the role of US signalling in inhibitory learning, they do point towards the US–CS interval being a more robust manipulation of inhibition than US signalling. The results, therefore, provide only partial support for the hypothesis that priming the US results in inhibitory learning and fail to support the hypothesis that associative priming (by signalling the US) and nonassociative priming (by the recent presentation of the US) have the same influence on US processing (Wagner, 1981).
